# Intracrine androgen biosynthesis and drug resistance

**DOI:** 10.20517/cdr.2020.60

**Published:** 2020-11-03

**Authors:** Trevor M. Penning, Irfan A. Asangani, Cynthia Sprenger, Stephen Plymate

**Affiliations:** ^1^Department of Systems Pharmacology & Translational Therapeutics, Perelman School of Medicine, University of Pennsylvania, Philadelphia, PA 19104, USA.; ^2^Department Cancer Biology, Perelman School of Medicine, University of Pennsylvania, Philadelphia, PA 19104, USA.; ^3^Division of Gerontology & Geriatric Medicine, Department of Medicine, University of Washington, Seattle, WA 98109, USA.; ^4^Geriatric Research Education and Clinical Center (GRECC), VA Puget Sound Health Care System, Seattle, WA 98108, USA.

**Keywords:** Prostate cancer, abiraterone acetate, enzalutamide, aldo-keto reductase 1C3, androgen biosynthesis

## Abstract

Castration-resistant prostate cancer is the lethal form of prostate cancer and most commonly remains dependent on androgen receptor (AR) signaling. Current therapies use AR signaling inhibitors (ARSI) exemplified by abiraterone acetate, a P450c17 inhibitor, and enzalutamide, a potent AR antagonist. However, drug resistance to these agents occurs within 12-18 months and they only prolong overall survival by 3-4 months. Multiple mechanisms can contribute to ARSI drug resistance. These mechanisms can include but are not limited to germline mutations in the AR, post-transcriptional alterations in AR structure, and adaptive expression of genes involved in the intracrine biosynthesis and metabolism of androgens within the tumor. This review focuses on intracrine androgen biosynthesis, how this can contribute to ARSI drug resistance, and therapeutic strategies that can be used to surmount these resistance mechanisms.

## Introduction

Prostate cancer is a leading cause of cancer in the U.S. male population resulting in 160,000 new cases per year and 34,000 deaths annually^[1]^. The seminal discoveries of Charles Huggins showed that surgical castration followed by adrenalectomy with replacement glucocorticoids led to remission and that the disease could be treated with androgen deprivation treatment (ADT)^[2,3]^. It is noteworthy that these studies implied that there could be two sources of androgens for the tumor to continue to grow, the testis and the adrenal glands. These observations led to the introduction of surgical castration and later leuprolide and other LH-RH agonists to cause a chemical castration as the mainstay of first-line ADT^[4-6]^. *R*-Biaclutamide (Casodex), an androgen receptor antagonist, is often added to this regimen^[7]^. Furthermore, *R*-biaclutamide can now be replaced with more potent AR antagonists such as enzalutamide^[8]^. Following, ADT, there is a period of remission, however, the cancer invariably returns to give rise to castration-resistant prostate cancer (CRPC)^[9,10]^.

CRPC is the lethal form of prostate cancer and is often detected by a rising serum prostatic-specific antigen (PSA). PSA is an androgen-dependent gene and this implies that despite castrate levels of circulating androgens, the disease remains dependent on androgen receptor (AR) signaling^[11]^. Castrate levels of androgens does not indicate an absence of androgens but only the presence of very low androgen levels in the circulation. Dependence on AR signaling can arise due to changes in the AR^[12,13]^. These changes include AR gene amplification^[14]^ so that it can respond to trace ligand, AR mutation so that the receptor becomes ligand promiscuous^[15-17]^, the appearance of splice variants that have lost their ligand binding domain and thus are constitutively active in the absence of ligand^[18]^, and phosphorylation to activate the receptor in the absence of ligand^[19,20]^. In addition, the tumor adapts to make its own androgens in response to castration. Traditional ADT does not address the adrenal source of androgens even though the work of Huggins identified that this was an important source. The ability of tumors to make their own steroid hormones is referred to as intracrine synthesis, and Labrie coined the phrase “intracrinology” to describe this process^[21,22]^. It is now recognized that enzymes and transporters involved in the regulation of ligands for steroid receptors determine the pre-receptor concentration of ligand for hormone action^[23,24]^.

Changes in AR and intracrine androgen biosynthesis are both adaptive responses to ADT. To attenuate these adaptive responses, androgen receptor signaling inhibitors (ARSI) were introduced and belong to two classes of agents. One class of agents are the potent AR antagonists enzalutamide and its second-generation analogs apalutamide and darolutamide^[25-28]^; and the other class of agents were the P450c17 (17α-hydroxylase/17,20-lyase) inhibitors, represented by abiraterone acetate^[11,29,30]^ and second-generation analogs galeterone^[31,32]^ and orterenol. Both classes of ARSIs improve progression-free survival but drug resistance emerges so the increase in overall survival may only be 3-4 months compared to standard ADT. ARSI drug resistance can involve increases in intracrine androgen synthesis mediated by transporters and enzymes, which determine the amount of ligand for the AR, and these mechanisms are the subject of this review.

## Intracrine androgen biosynthesis

In a castrate environment, the major source of precursors of potent androgens in the tumor are those derived from the adrenal glands. The adrenal androgens of interest are the C19 steroids dehydroepiandrosterone (DHEA), DHEA-SO_4_, Δ^4^-androstene-3,17-dione (Δ^4^-AD), and the 11-oxygenated androgens (11β-hydroxy-Δ^4^-AD and 11-oxo-Δ^4^-AD)^[33]^. Of these, DHEA-SO_4_ is the dominant serum steroid, and is present in a huge excess compared to all other circulating steroids^[21,34]^.

In addition to the contribution from adrenal steroids, intracrine steroid hormone biosynthesis could start from cholesterol using the side-chain cleavage enzyme (P450Scc) to produce pregnenolone. Tumor P540c17 would then convert pregnenolone to DHEA. While changes in transcripts for these enzymes have been observed in prostate cancer cell lines and in CRPC tumor biospecimens^[23]^, there is no compelling evidence to support the conversion of C27 steroids (cholestanes) into C19 steroids (androgens) by flux measurements^[35-37]^. Moreover, the large amount of DHEA-SO_4_ that exists in the systemic circulation with and without P450c17 inhibition makes it seem unlikely that the tumor needs to adapt to castration or ADT to make its own DHEA^[34]^.

**Figure 1:**
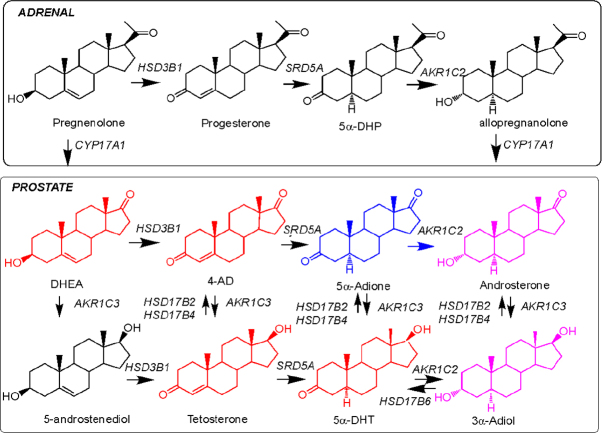
Intracrine androgen biosynthesis. Top panel shows reactions that occur in the adrenal reticularis. Bottom panel shows the conversion of adrenal steroids into the potent androgens testosterone and 5α-DHT in human prostate. The classical or canonical pathway is shown in red; the 5α-adione pathway is shown in blue; the backdoor pathway from allopregnanlone is shown in purple; the alternative pathway from 5-androstenediol is also shown. All enzymes are listed in italics by their gene names as follows: *AKR1C1*, 3α(20α)-hydroxysteroid dehydrogenase; *AKR1C2*, type 3 3α-hydroysteroid dehydrogenase; *AKR1C3*, type 5 17β-hydroxysteroid dehydrogenase; *CYP17A1*, 17α-hydroxylase17/20 lyase; *HSD3B1*, type 1 3β-hydroxysteroid dehydrogenase; *HSD17B2* and *17HSD17B4*, type 2 and type 4 17β-hydroxysteroid dehydrogenase; *HSD17B6*, type 6 17β-hydroxysteroid dehydrogenase and retinol dehydrogenase; and *SRD5A1/2*, type 1 and type 2 steroid 5α-reductase

For DHEA-SO_4_ to be utilized by prostate tumors, two events have to take place. First, the anionic steroid needs to be transported by an organic anion transporter protein of the *SLCO* gene family and, second, steroid sulfatase (STS) needs to remove the sulfate group to generate free DHEA [Figure 1]. DHEA is then converted to Δ^4^-AD by the bifunctional enzyme HSD3B1, which has both 3β-hydroxysteroid dehydrogenase activity and ketosteroid isomerase activity. Δ^4^-AD represents a junction point; it can be converted by the canonical pathway to testosterone (T) by type 5 17β-hydroxysteroid dehydrogenase (AKR1C3) and then reduced by steroid 5α-reductase (SRD5A1 and SRD5A2) to 5α-dihydrotestosterone (DHT) as demonstrated in clinical specimens^[38,39]^. Alternatively, Δ^4^-AD can be converted by steroid 5α-reductase (SRD5A1 and SRD5A2) to yield 5α-androstane-3,17-dione, which is then reduced by AKR1C3 to yield DHT. The latter pathway bypasses T altogether and has been demonstrated in prostate cancer cell lines and xenografts as being the preferred pathway^[40]^ and was confirmed to occur in hormone refractory cancer^[41]^. However, in CRPC patients and soft tissue metastasis the ratio of T:DHT favors formation of T which can be explained by a down regulation of SRD5A2 and up-regulation of AKR1C3^[42]^. DHEA is also reduced to 5-androstene-3β,17β-diol by AKR1C3, and then converted to T by HSD3B1. Finally, there is a backdoor pathway in which 5α-androstane-3,17-dione is reduced to androsterone by AKR1C2, and then further reduced by AKR1C3 to 5α-androstane-3α,17β-diol^[43]^. The diol is then oxidized by HSD17B6 to DHT^[44,45]^.

**Figure 2:**
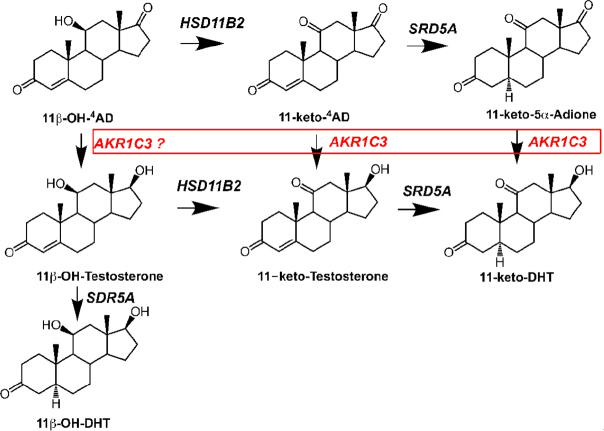
Conversion of 11-oxygenated androgens to 11-keto-Testosterone and 11-keto-DHT. The pathway from adrenal 11β-hydroxy-^4^AD and 11-keto-^4^AD to 11-keto-testosterone and 11-Keto-DHT, respectively is shown. The enzymes implicated in this conversion in the prostate are shown. All enzymes are listed in italics by their gene names. *AKR1C3*, type 5 17β-hydroxysteroid dehydrogenase; *HSD11B2*, type 2 11β-hydroxysteroid dehydrogenase; and *SRD5A1/2*, type 1 and type 2 steroid 5α-reductase

The canonical pathway described above can also convert 11-keto-Δ^4^-AD to produce 11-keto-T and 11-keto-DHT. In addition, the bypass pathway could convert 11-keto-Δ^4^-AD to 11-keto-5α-androstane-3, 17-dione on route to 11-keto-DHT and thereby bypass 11-Keto-T altogether^[46-48]^ [Figure 2]. Transfection studies in HEK-293 cells and studies with recombinant AKR1C3 indicate that 11-keto-Δ^4^-AD and 11-keto-5α-androstane-3,17-dione are the preferred substrates for AKR1C3 over Δ^4^-AD and 5α-androstane-3,17-dione^[49,50]^. Both 11-keto-T and 11-keto-DHT are equipotent to T and DHT in AR driven luciferase reporter gene assays^[46]^. These pathways underscore the importance of AKR1C3 in all pathways to form potent ligands for the AR.

By contrast, the related AKR1C enzymes, AKR1C1 and AKR1C2 play important roles in the inactivation of DHT^[51]^. AKR1C1 works predominately as a 3-ketosteroid reductase on DHT leading to the formation of 5α-androstane-3β,17β-diol, a pro-apoptotic ligand for estrogen receptor β^[52]^. AKR1C2 works predominately as a 3-ketosteroid reductase on DHT leading to the formation of the inactive androgen 5α-androstane-3α,17β-diol^[53]^. Similar reactions are possible with 11-keto-DHT^[50]^. As described above, HSD17B6 is involved in the back conversion of 5α-androstane-3α,17β-diol to DHT, and compelling evidence exists that by working in opposite directions, AKR1C2 (3-ketosteroid reductase) and HSD17B6 (3a-hydroxysteroid oxidase) work as the molecular switch that determines DHT ligand access to the AR in the normal and diseased prostate^[53]^. HSD17B2 and HSD17B4 by working as 17β-hydroxysteroid oxidases convert T and DHT to their inactive counterparts, *e.g.*, Δ^4^-AD and 5α-androstane-3,17-dione, respectively, and are implicated in the inactivation of these hormones^[54,55]^. The 5α-androstanediols once formed by AKR1C1 and AKR1C2, can then be glucuronidated by UGT family members UGT2B15 and UGT2B17^[56,57]^. Both UGT2B15 and UGT2B17 expression have been shown to be increased in CRPC.

## Androgen biosynthesis and metabolism and drug resistance

**Figure 3:**
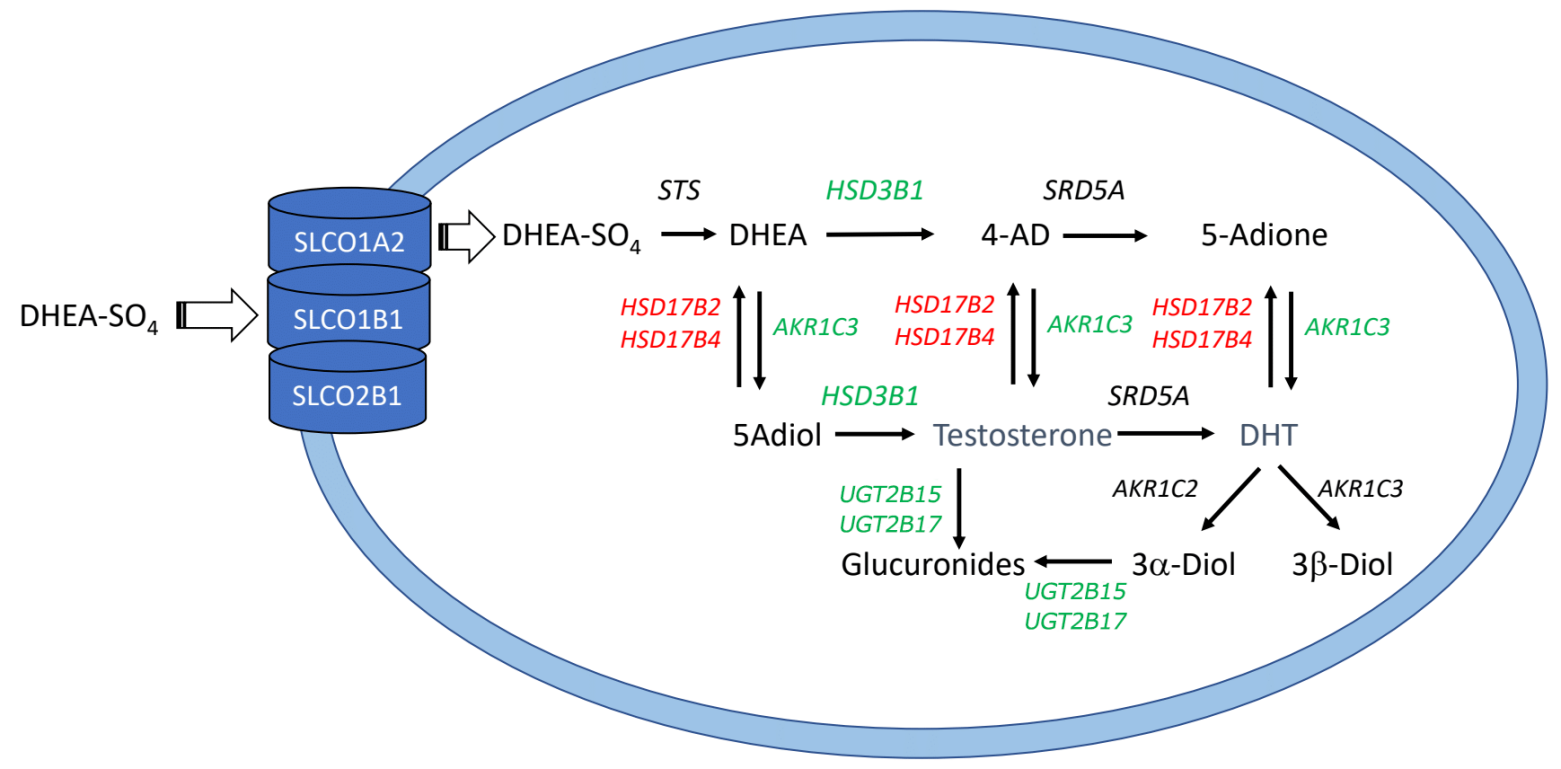
Mechanisms of drug resistance to androgen receptor signaling inhibitors (ARSI). Following uptake of DHEA-SO_4_ into the prostate by SLCO transporters a number of enzymes with either increased expression or germline mutations that result in increased activity/stability may contribute to ARSI drug therapy resistance (shown in green); similarly, a number of enzymes with either decreased activity or germline mutations that decrease activity/stability may contribute to drug resistance (shown in red). AKR1C3 may be an exception in that it is highly upregulated by androgen deprivation, and nsSNPs in conserved positions may reduce its activity. All enzymes are listed in italics by their gene names as described in the legends to Figures 1 and 2

Intracrine androgen biosynthesis has an important role in resistance to ARSI. Abiraterone acetate and other P450c17 inhibitors leave behind a significant reservoir of DHEA-SO_4_ in the serum following leuprolide treatment^[58,59]^, while enzalutamide will have no effect on adrenal androgens following chemical or surgical castration. For example, the amount of DHEA-SO_4_ that remains even after combined leuprolide and abiraterone treatment is 4,000 times higher than the castrate amounts of T achieved. Resistance to ARSI can be enhanced when there is either upregulation or activating germline mutations in SLCO transporters or androgen biosynthesis genes or down-regulation or inactivating germline mutations in androgen metabolizing genes that result in elevated intra-tumoral T and DHT [Figure 3]. Thus, the serum levels of DHEA-SO_4_ that exist in patients on ARSI coupled with increased intracrine formation of T and DHT combined with increased AR expression create a “perfect-storm” for drug resistance.

### Genes required for androgen biosynthesis

#### *SLCO* genes

Transcripts of *SLCO1A2, SLCO1B1* and *SLCO1B2* are increased 4-fold, 2.5-fold, and 1.74-fold respectively in LNCaP and 22Rv1 prostate cancer cells grown under conditions of androgen depletion to mimic ADT. Increased *SLCO* expression led to increased cellular uptake of [^3^H]-DHEA-SO_4_ and increased cellular proliferation stimulated by DHEA-SO_4_. The cellular proliferation in response to DHEA-SO_4_ was attenuated by *SLCO1A2* knockdown, indicating the requirement of these transporters to facilitate intra-tumoral androgen biosynthesis^[60]^. *SLCO1B1-* and *SLCO2B1*-expressing prostate cancer xenografts showed a 3.9-fold and 1.9-fold increase in tumor accumulation of DHEA-SO_4_, respectively^[61]^. *SLCO* genes are also overexpressed in CRPC metastases. When 1,309 Caucasian prostate cancer patients were genotyped, nsSNPs in *SLCO* were associated with a poor progression-free survival^[24]^. For example, three SNPs in *SLCO2B1* rs1242149, [935G>A; Arg312Gln], rs1789693 (intronic), and rs1077858 (intronic) were associated with decreased time to progression on ADT (*P* < 0.05). When the *SLCO2B1* SNP variant rs1242149 (935G>A; Arg312Gln) was transfected into LNCaP cells, transfected cells exhibited higher maximal DHEA-SO_4_ accumulation when compared with the wild-type allele^[62]^. It is predicted that variants in the *SLCO* genes may serve as predictors of DHEA-SO_4_ uptake and ARSI resistance (see Table 1 for a summary of the common nsSNPs in genes involved in androgen biosynthesis and metabolism in the human prostate).

**Table 1:** Common nsSNPs in genes involved in intracrine androgen biosynthesis and metabolism

Gene	Accession No.	Nucleotide change	Minor allelic frequency	Amino-acid change	Change in function
*SLCO2B1*	rs12422149	935G>A	0.2099	Arg312Gln	
*HSD3B1*	rs1047303	1245 A>C	0.2450	Asn367Thr	Increase protein stability
*AKR1C1*	rs149693250	116 A>G	0.0046	Lys39Arg	
	rs540512862	440 C>T	0.0022	Thr147Ile	
	rs139588200	509 G>A	0.0028	Arg170His	
	rs142200840	840 C>A	0.0076	Asn280Lys	
	rs201500205	891 Ter	0.0016	Tyr323ns	
*AKR1C2*	rs149358453	68 C>T	0.0002	The23Ile	
	rs2854482	137 T>A	0.0649	Phe46Tyr	
	rs147648222	209 C>T	0.0034	Ala70Val	
	rs142672563	211 G>T	0.0026	Asp71His	
	rs145967531	364 G>A	0.0026	Val122Ile	
	rs139521690	355 C>A	0.0000	Pro119Thr	
	rs114734	515 T>A	0.0002	Leu172Gln	
	rs2518043	553 A>G	ND	Lys185Glu	
	rs782195517	772 C>T	0.064	Arg258Cys	
	rs539972269	837 G>C	0.001	Gln279His	
*AKR1C3*	rs12529	15 C>G,T	0.4203	His5Gln	Decrease protein activity
	rs1155177	230 A>G	0.0367	Glu77Gly	Decrease protein activity
	rs14888604	253 C>T	0.0000	Leu85Phe	
		253 C>G		Lue85Val	
	rs12387	312 G>C	0.1518	Lys104Asp	Decrease protein activity
	rs34186955	538 C>T	0.0086	Pro180Ser	
	rs61730879	548 A>G	0.0026	Lys183Arg	
	rs199934766	595 C>T	0.0004	Arg199Trp	
	rs139146411	596 G>A	0.0004	Arg199Gln	
	rs62621365	772 C>T	0.0325	Arg258Cys	Decrease protein activity
	rs1804059	879 G>T		Met293Ile	
*SRD5A2*	rs763681675	7 G>C	ND	Val3Ile	
	rs766177308	42 C>A	ND	Ser14Arg	
		G>A		Ala49Thr	Increase protein stability
	rs1465766670	187G>A	ND	Val63Met	
	rs753942411	354C>G	ND	Phe118Leu	
	rs759561106	433C>T	ND	Arg145Trp	Reduced affinity for NADPH
	rs756405261	513G>T	ND	Arg171Ser	Reduced affinity for NADPH
		G>A		Gly183Asp	
	rs774564339	565G>A	ND	Val189Ala(Leu)	
		G>A		Gly191Glu	
		T>C		Leu221Pro	
		T>C		Leu226Pro	Increase protein stability
	rs142602296	686 T>C	ND	Phe229Ser	
	rs145712014	734 C>A	0.0006	Ser245Tyr	Reduced affinity for NADPH
		C>T		Ala248Val	
*UGT2B15*	rs1902923	G>T	0.32	Asp85Tyr	
	rs4148269	1568 A>C	0.450	Lys523Thr	

nsSNPs: non-synonymous single nucleotide polymorphisms

#### *STS* gene

There is only one steroid sulfatase gene (*STS*) which is required for the deconjugation of DHEA-SO_4_ to from free DHEA for downstream androgen biosynthesis. Knockdown of *STS* in castrate-resistant prostate cancer cell lines demonstrates the dependency of T and DHT synthesis on active *STS*. However, inhibition of STS for the treatment of CRPC is not a good strategy, since STS deficiency leads to ichthyosis which has been observed naturally in X-linked ichthyosis^[63]^.

#### *HSD3B1* gene

The transcriptional regulation of *HSD3B1* has been examined in multiple metastatic prostate cancer cell models. *HSD3B1* is induced by androgens in VCaP, CWR22Rv1, LNCaP, and LAPC4 models over 72 h but is then attenuated after 120 h. Thus, the enzyme does not appear to be regulated by androgen deprivation^[64]^.

A common 1245 A→C missense- single nucleotide polymorphism in *HSD3B1* (rs1047303) results in the amino acid change Asn367Thr. This mutation does not change the kinetic properties of the enzyme but leads to a more stable protein that is resistant to degradation suggesting that the variant will lead to increased intra-tumoral androgen biosynthesis and ARSI drug resistance^[65]^. Although it is not possible to determine how this mutation affects protein stability without a crystal structure for the enzyme, clinical studies show that the adrenal permissive mutation 1245 A→C is associated with inferior outcomes to ADT^[66,67]^.

The ARSI abiraterone has a 3β-hydroxyl- Δ^5^-ene in its structure and can be metabolized to Δ^4^-abiratreone by HSD3B1, which is then converted to 5α-abiraterone by SRD5A1/SRD5A2. Each of these metabolites have their own activity. Δ^4^-Abiraterone has anti-tumor activity since it inhibits P450c17, HSD3B1, and is an antagonist of the AR^[68]^, leading to the concept that abiraterone acetate may be a pro-drug and that Δ^4^-abiraterone is the active metabolite^[69]^. By contrast, 5α-abiraterone will activate the AR, potentially driving cancer progression. Thus, the mutant *HSD3B1* allele can contribute to ARSI drug resistance by this mechanism.

#### *AKR1C3* gene

*AKR1C3* is one of the most highly expressed steroidogenic genes in CRPC; this has been seen by RT-PCR and Affymetrix microarray^[70]^. AKR1C3 is upregulated by androgen deprivation in prostate cancer cells^[71]^, xenografts^[71]^, and patient tumor samples^[72-74]^. Castration also induces up-regulation of AKR1C3 in an orthotopic VCaP human prostate cancer xenograft and leads to tumor growth^[74]^. Ten or more separate studies have replicated the finding that AKR1C3 is overexpressed in CRPC^[38,39,70-73,75,76]^. One such study showed AKR1C3 overexpression in primary lesion re-biopsies at the time of metastatic disease^[77]^. Consecutive prostate cancer specimens revealed increased AKR1C3 expression during progression to CRPC^[78]^. It is estimated that approximately 30% of CRPC patients will overexpress this enzyme. AKR1C3 has since been proposed as a biomarker for the CRPC. Experiments aimed at determining the mechanism of AKR1C3 over-expression have implicated the involvement of the TMPRSS2-ERG fusion protein in its regulation^[75]^. The TMPRSS2-ERG fusion protein arises in late-stage disease based on Gleason grade, where ERG acts as a transcription factor to increase AKR1C3 expression and remove the repressive effect of the AR on the *AKR1C3* promoter. In this mechanism, ERG binds to the *AKR1C3* promoter to induce gene transcription to increase T or DHT production which in turn increases TMPRSS2-ERG expression to induce AKR1C3 expression in a “feed-forward” manner^[75]^.

Treatment of prostate cancer cells with abiraterone or enzalutamide all increase AKR1C3 expression consistent with androgen-dependent repression of the *AKR1C3* gene^[79,80]^. C4-2B prostate cancer cells when grown in the presence of either abiraterone or enzalutamide over-express AKR1C3 and are resistant to growth inhibition by these drugs. The drug-resistant cell lines are, however, sensitive to growth inhibition in colony formation assays in soft agar and in xenografts to the AKR1C3 competitive inhibitor indomethacin^[79,80]^. The efficacy of indomethacin to improve progression-free survival and overall survival is being tested in a clinical trial in patients that progress on enzalutamide (NCT02935205 in clinical trials.gov), and in patients that progress on abiraterone acetate (NCT0254990 in clinical trials.gov). Similarly, AKR1C3 inhibitors could be used to surmount resistance to apalutamide and darolutamide^[81]^, discussed later in this paper.

There are 14 *AKR1C3* non-synonymous single nucleotide polymorphisms (nsSNPs) with varying global frequencies. The top-most frequently occurring variant (His5Gln) occurs in over 50% of the global population with an occurrence of 43% in African Americans. Lys104Asp, Glu77Gly, Arg258Cys have minor allelic frequencies of 15%, 3.7%, and 3.3%, respectively. All of these mutations were examined for their ability to conduct 17-ketosteroid reduction of the aromatase inhibitor exemestane, and each gave a significant reduction in catalytic efficiency that was 17-170 fold lower than the wild-type enzyme^[82]^. The remainder of the nsSNPs have a minor allelic frequency (MAF) of > 1%. It is unknown if these nsSNPs are enriched in prostate cancer patients. These nsSNPs can be mapped to the crystal structure of AKR1C3 and do not reside in the cofactor binding site, steroid binding cavity, or at the active site. AKR1C3 has a a/b_8_-barrel structure which is evolutionarily conserved across phyla in the AKR gene superfamily. In *AKR1C3* there are seven nsSNPs that are in amino acids that are evolutionarily conserved in the structure (Leu85Phe; Pro180Ser; Lys183Arg; Arg199Trp; Arg199Gln; Arg258Cys; Met293Ile). These amino acids are conserved since they likely maintain protein folding and therefore mutations in these amino acids likely affect protein stability. It is predicted that carriers of these allelic variants may be more responsive to ARSI therapy.

#### *HSD17B6* gene

The *HSD17B6* gene has a number of aliases, e.g., RL-HSD, and actually functions as a 3α-hydroxysteroid dehydrogenase and epimerase rather than a 17β-HSD. It was identified as the major oxidoreductase responsible for the back conversion of 5α-andostane-3α,17β-diol to DHT in prostate cancer^[44,45]^. Few SNPs have been reported in this gene to date.

#### *SRD5A* genes

Abnormal *SRD5A2* deficiency [46, XY SRD5A2 an autosomal recessive disorder of sex development (DSD)] has been well documented and associated with pseudo-hermaphroditism, lack of male pattern baldness, and an atrophied prostate gland^[83]^. There are more than 114 different mutations in the *SRDA2* gene (including missense/nonsense mutations, deletions, insertions, and indels). However, less than 20 have been expressed to determine the consequences of these mutations on enzyme function. The protein is predicted to have two putative functional domains, an NADPH cofactor binding domain and a steroid binding domain. Ser14Arg, Arg145Trp, Arg171Ser, Phe229Ser, Ser245Tyr result in either complete loss of enzyme activity or seriously diminished enzyme activity. Arg145Trp, Arg171Ser, and Ser245Tyr are predicted to have diminished affinity for NADPH^[84]^. Many SNPs have been associated with prostate cancer and lead to the following amino acid changes: Val3Ile, Ala49Thr, Val63Met, Phe118Leu, Gly183Asp, Val189Ala, Gly191Glu, Leu221Pro, Leu226Pro, and Ala248Val, and have been kinetically characterized. Of these, Ala49Thre and Leu226Pro had significantly higher utilization ratios (V_max_/*K*_m_)^[85]^. The loss-of-function mutations in prostate cancer patients are likely to make the tumor more dependent on T rather than DHT, and may, therefore, have a neutral effect in response to ARSI therapy, and their occurrence is unlikely to be a major mechanism of drug resistance.

A third steroid 5α-reductase gene (*SRD5A3*) has also been identified. However, there has been controversy as to whether this reductase is really involved in the conversion of T to DHT. There is strong evidence that this gene instead encodes for a polyprenol reductase required for the synthesis of dolichol, and that mutations in this gene lead to a congenital glycosylation disorder^[86]^.

### Genes required for androgen metabolism

#### *AKR1C1* and *AKR1C2* genes

The *AKR1C1* and *AKR1C2* genes are highly induced by the Nrf2-Keap 1 pathway due to the presence of an antioxidant response element in their promoters^[87]^. However, the effect of oxidative stress and electrophilic induction of these genes on androgen metabolism has yet to be described. Increased expression of AKR1C1 and AKR1C2 in prostate cancer would lead to the elimination of DHT and may favor the unexpected high ratio of T:DHT in metastatic prostate cancer specimens. There are a several nsSNPs in AKR1C1: Lys39Arg, Thr147Ieu, Arg170His, Asn280Lys, and Thre323ns, with MAF less than 0.0076. The effects of these mutations on enzyme function have not been determined.

There are a number of nsSNPs in *AKR1C2* (Phe46Tyr, Ala70Val, Asp71His, Val122Ile, Pro119Thr, Leu172Gln, Lys185Glu, Gln279His, and Arg258Cys). Three of these (Pro119Thr, Lys185Glu, and Arg258Cys) are in evolutionarily conserved residues, whereas Arg258Cys has a MAF of 0.064. In addition, Phe46Tyr has a MAF of 0.0649, but the MAF of the remainder are > 0.0034. The effect of some of these allelic variants on the *in vitro* metabolism of DHT has been examined. Unfortunately, the authors examined the effect of these variants following expression in Sf9 insect cell lysates and used a catalytic inactive mutant Tyr55Phe as a control. Under these conditions, a significant background turnover of DHT was noted in the presence of the catalytically inactive Tyr55Phe mutant making it difficult to interpret these data^[88]^. The authors concluded that Phe46Tyr (0.0649 MAF) and Leu172Gln (not in NCBI) reduced the apparent V_max_ and that Leu172Gln, Lys185Glu and Arg258Cys all reduced the apparent *K*_m_. However, their effect on the utilization ratio V_max_/*K*_m_ for reduction of DHT by these variants was modest and varied by only 2- to 3-fold.

#### *HSD17B2* gene

*HSD17B2* expression is reduced in prostate cancer patients, and consistently a *HSD17B2* gene deletion was found in both primary and metastatic prostate cancer. In xenograft models over-expression of HSD17B2 suppressed androgen-induced cell proliferation and xenograft growth, consistent with its enzyme activity to inactivate 17β-hydroxyandrogens. Mechanisms responsible for this reduced expression included DNA methylation and mRNA alternative splicing. Two new catalytic-deficient shorter isoforms generated by alternative splicing were found to bind to the full-length enzyme promoting its degradation and are involved in the functional silencing of HSD17B2^[89]^. It is unknown if this alternative splicing mechanism occurs more frequently in ARSI drug resistance.

#### *HSD17B4* gene

*HSD17B4* expression can increase in CRPC and predicts poor prognosis which appears counterintuitive based on its role to inactive androgens. However, there are five alternatively spliced isoforms, and only isoform 2 inactivates T and DHT, and it is this isoform that is suppressed in CRPC. Genetic knockdown of *HSD17B4* isoform 2 increases T and DHT to stimulate the AR and CRPC development in xenograft models^[55]^. It is unknown if reduced HSD17B4 expression contributes to ARSI drug resistance.

#### *UGT2B15* and *UGT2B17* genes

*UGT2B15* and *UGT2B17* are actively involved in the elimination of hydroxyandrogens as glucuronides from prostate cancer cells^[56]^. These genes are characterized by common polymorphisms and copy number variants that affect enzymatic activity and expression. Whole-gene deletions in *UGT2B15* and *UGT2B17* are observed in 27% and 13.5% of Caucasians and over 50% have a deletion of one of these genes. The nsSNP in *UGT2B15* (Asp85Tyr, rs1902923 G.T) increases V_max_ by several fold and has a MAF of 0.32. Both these changes have been associated with increased prostate cancer risk. The expression of *UGT2B17* is inversely correlated with activation of the full-length AR receptor consistent with maintaining a homeostasis of AR signaling in normal prostate and castration sensitive prostate cancer. However, castration resistance leads to the expression of the constitutively active AR-V7 splice variant which upregulates *UGT2B17* expression^[90-92]^. Although this seems counterintuitive, increased expression of *UGT2B17* induced by AR-SV could be part of a coordinated response to make the tumor remain dependent on AR signaling in the absence of ligand^[91]^. Additional *UGT2B15* polymorphisms (namely rs4148269, rs3100 (3’-UTR) rs9994887 (upstream), rs13112099 (upstream), rs7686914 (upstream), and rs7696472 (upstream) have been associated with an increased risk of PCa in a multiethnic study^[93]^.

## Non-classical properties of steroidogenic enzymes

**Figure 4:**
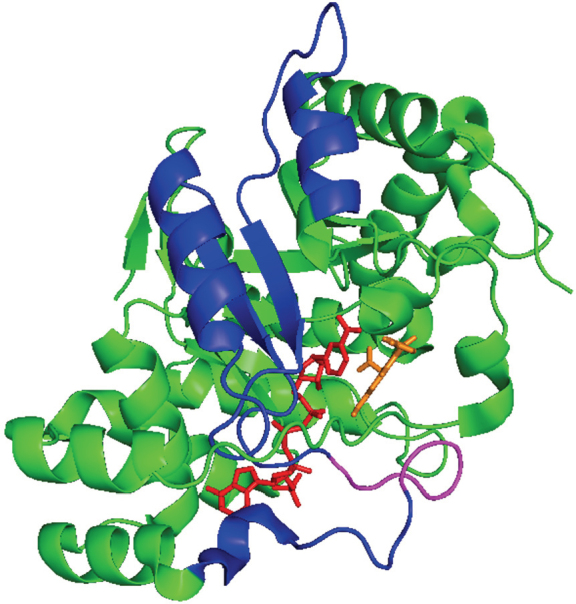
Putative Coactivator Domain of AKR1C3. Ribbon diagram of the AKR1C3•NADP^+^•Indomethacin complex (taken from PDB ID: 3UGB) showing the putative coactivator domain in the enzyme. Protein fold (green); NADP+ stick representation (red); indomethacin stick representation (orange); blue corresponds to amino acids 171-237; the disordered loop that may bind to the N-terminus of the AR (magneta). Cartoon prepared in PyMol

Several of the steroidogenic enzymes have other properties that contribute to their role in ARSI drug resistance. One such enzyme is AKR1C3, that can act as a coactivator of the AR^[94]^. AR coactivators are AR-interacting proteins that amplify AR-dependent gene transcription in the presence of ligand^[95]^. AKR1C3 is a selective coactivator of AR, having no effect on the transcription mediated by other nuclear receptors. Furthermore, AKR1C3 acts as coactivator on AR with an activity comparable to SRC-2^[94]^. The co-activator domain of AKR1C3 maps to amino acid residues 171-237 which is distinct from the active-site. This region contains a putative p160 coactivator peptide of LxxIL buried within α-helix 5, were the consensus sequence to bind to the ligand binding domain of AR is LxxLL^[96]^. Although this motif can bind to the AF-2 region of the ligand binding domain of the AR, this interaction is out competed by a N/C interdomain interaction involving the FxxLF motif in the N-terminus of AR^[96,97]^, leaving the identity of the coactivator peptide domain of AKR1C3 uncertain. The LxxLL motif present in AKR1C3 is also conserved within the AKR1C1 and AKR1C2 sequence and these proteins do not have coactivator properties. Comparison of the 171-237 region in all three enzymes shows a difference in amino acid residues in these enzymes. In AKR1C3, the sequence corresponds to ^222^QRDKRW^227^ whereas in AKR1C1 and AKR1C2 the sequence corresponds to ^222^HREEPW^227^. Mapping this region to the AKR1C3 crystal structure shows that this peptide is in a disordered loop. It is likely that this loop interacts with the NTD pf the AR [Figure 4].

Confocal microscopy and proximity ligation assays have validated the interaction between AKR1C3 and the AR, and the co-translocation of both proteins to the nucleus. While many different coactivators exist for the AR, AKR1C3 has a unique property of being up-regulated by androgen deprivation in CRPC. AKR1C3 stabilizes AR-V7, a major AR-SV observed in clinical specimens^[98]^. In addition, AKR1C3 binds and stabilizes the ubiquitin ligase Siah-2, inhibiting its degradation, thereby enhancing Siah-2-dependent down-regulation of the AR corepressor NCoR in prostate cancer cells^[99]^.

Another function of AKR1C3 is its ability to be the dominant prostaglandin F2α synthase in humans. This enzyme function leads to the formation of PGF_2α_ epimers that can activate the FP1 receptor^[100]^. Activation of the receptor leads to activation of the MAPK pathway and phosphorylation of PPARγ, leading to its inactivation. Inactivation of PPARγ is considered a pro-proliferative signal since it prevents cellular differentiation. The involvement of this pathway in CRPC remains to be determined.

## Inhibitors of androgen biosynthetic enzymes for CRPC

Drug resistance to ARSI could be attenuated by inhibitors of HSD3B1, AKR1C3, HSD17B6, and SRD5A2/A1. However, there are no inhibitors that have the desired selectivity for HSD3B1 over HSD3B2, and inhibition of these enzymes would block all steroid hormone biosynthesis. Both finasteride (SRD5A1 inhibitor) and dutasteride (dual SRD5A1/SRD5A2 inhibitor) have been shown in chemoprevention trials to give rise to a reduction in tumor incidence but also cause an increase in high-grade disease as determined by Gleason grade^[101,102]^. There has been much debate as to whether this was due to increased patient monitoring and frequency of needle biopsy, despite the increase in high-grade disease being small. Nonetheless, the FDA has issued black-box warnings for their use in prostate cancer therapy. Inhibitors of HSD17B6 appear to be in their infancy. Thus, the focus of our discussion will be on inhibitor development for AKR1C3.

### AKR1C3 inhibitors

A large number of small-molecule AKR1C3 inhibitors have been developed for the potential treatment of CRPC as a monotherapy and to surmount ARSI drug resistance. Effective compounds need to show potency and selectivity for the target. Compounds must not inhibit AKR1C1 or AKR1C2 which would inactivate DHT. This can be challenging since the AKR1C enzymes exhibit 86% sequence identity and have similar crystal structures^[103]^.

AKR1C3 inhibitors that have been developed are both non-steroidal and steroidal in nature. The non-steroidal inhibitors include re-purposed NSAID analogs that no longer inhibit Cox-1 and Cox-2, based on *N*-phenylanthranilic acid, indomethacin and *R*-naproxen analogs^[104-106]^, and their bioisoteres^[107]^, as well as 3-(3,4-dihydroisoquinolin-2(1H)-ylsulfonyl)benzoic acids^[108]^, caffeic acid phenethyl esters^[109]^, berberine analogs^[110]^, 1-(4-(piperidin-1-ylsulfonyl)phenyl)pyrrolidin-2-ones^[111]^, 2,3-diarylpropenoic acids^[112]^, and ASP9521 (developed by Astellas)^[113,114]^, GTx-560 (developed by GTx-therapeutics)^[94]^; the natural product analogs based on baccharin^[115]^ and amaryllidaceae alkaloids^[116]^; and finally the steroidal based analogs developed by Bayer, e.g., BAY1128688, that are proprietary. Crystal structures of AKR1C3•NADP^+^•Inhibitor complexes exist for many of these agents, and there is a good structural basis for their mode of inhibition. Carboxylic acid analogs or those that contain an organic anion form a counter ion with the catalytic tetrad in which Tyr55 has TyrOH_2_^+^ character. Depending on the appendages to the anion, side chains can occupy three different pockets: the steroid binding cavity, sub-pocket (SP)1 (lined by Ser118, Phe306, Leu308, and Tyr319), SP2 (lined by Leu129, Phe306, and Phe311), and SP3 (lined by Tyr24, Ser221, Ser217, Gln222, Phe306)^[117]^. A recent patent review on many of these agents was published by our group^[118]^. Rather than review all these agents a few highlight points will be made.

First, indomethacin has made it into clinical trial for patients who progress on enzalutamide. Due to the gastrointestinal side effects associated with chronic use of this NSAID, indomethacin analogs, that are more potent and selective for AKR1C3 than the parent compound and that do not inhibit the COX isozymes, offer promise to replace indomethacin^[106]^.

Second, ASP9521 is an AKR1C3 inhibitor that went into a phase 1/2 clinical trial and was found to be well tolerated but without efficacy^[113,114]^. However, there were several reasons why this trial failed. Of the small number of patients enrolled 6/13 failed to complete their drug regimen. Patients were excluded from the trial if they had been on abiraterone or *R*-biaclutamide before the commencement of the trial but these would be the very treatments that would upregulate AKR1C3. Moreover, there was no determination of whether the patient tumors were AKR1C3 positive, even though there is an IHC grade monoclonal Ab available^[119]^.

Third, the baccharin anlogs (Kv37) are the only agents that have been shown to have synergistic anti-proliferative effects with enzalutamide in vitro, where the combination index shows up to a 200-fold synergistic effect^[115]^. However, this does not mean that other monofunctional AKR1C3 inhibitors will not behave the same way. They have not yet been screened in drug combinations.

Fourth, steroidal analogs, e.g., BAY1128688, run the risk of also inhibiting other steroid-transforming enzymes or steroid receptors. It is noteworthy that BAY1128688 went into a phase 2 clinical trial for endometriosis that was halted due to hepatic toxicity^[120]^. However, it is not clear whether the compound was counter screened against other AKRs, including AKR1C4 and AKR1D1 that are essential for bile acid biosynthesis^[103]^.

Fifth, the *N*-phenylaminobenzoates based on *N*-phenylanthranilic acids gave rise to the *N*-naphthylaminobenzoate (BMT4-158) that is a “first-in-class” bifunctional AKR1C3 inhibitor and AR antagonist^[121]^. In a recent head to head comparison of this agent with ASP9521 and GTx-560, BMT-4-158 out-performed these agents since it was the only compound that acted as a competitive inhibitor in AR binding assays and displaced R1881 in AR competitive binding assays^[122]^.

Sixth, GTx-560 is a “first-in-class” bifunctional AKR1C3 inhibitor that competitively inhibits the enzyme function of AKR1C3 but also blocks its coactivator function on the AR^[94]^. The agent had anti-tumor activity in xenograft models of prostate cancer, and it is unclear why this compound was not taken into clinical trials. This compound and its analogs offer promise to block two mechanisms of ARSI resistance, intra-tumoral androgen biosynthesis, and AR coactivation.

It is apparent that a large number of competitive inhibitors of AKR1C3 exist that have the desired potency and selectivity, however, if used as monofunctional agents, it is likely that other mechanisms of drug resistance will emerge, e.g., the appearance of AR splice variants (AR-SVs). Bifunctional inhibitors BMT4-158 and GTx-560 offer the promise of blocking intra-tumoral androgen biosynthesis and AR transactivation.

## Alternative mechanisms of drug resistance

Upregulation of AKR1C3 in response to ADT and its contribution to drug resistance to ARSI is only one mechanism of resistance to these agents. Multiple mechanisms may contribute to the drug resistance phenotype, they may not be mutually exclusive, they may also synergize with each other, and different mechanisms may contribute differently based on the patient. One mechanism involves mutations in other steroidogenic genes such as HSD3B1, where the stability mutant enabled increased formation of Δ^4^-AD and 5α-DHT^[65]^. When combined with upregulation of AKR1C3 these changes could lead to increases in steroidogenesis that is greater than that achieved by either change alone. A second mechanism involves the appearance of AR-SVs as the disease emerges^[18,123]^. AR-SVs have lost their ligand binding domain and some of these AR-SVs are constitutively active in the absence of ligand. AR-V7 is stabilized by AKR1C3 so that overexpression of the latter will increase the steady state concentration of AR-V7^[98]^. AR-V7 is one of the most prominent AR-SVs, leading to attempts to develop clinical assays to detect this variant in biospecimens to determine whether ARSI drug therapy should be discontinued^[124-126]^. A third mechanism involves phosphorylation of the AR-MED1 transcriptional complex mediated by cyclin-dependent kinase 7 (CDK7)^[127]^. Phosphorylation on AR Ser81 is sufficient to activate the AR in the absence of ligand^[128]^ and may contribute to resistance to enzalutamide, darolutamide, and apalutamide. It is unknown whether AKR1C3 could act as a coactivator of phosphorylated AR. A fourth mechanism involves the hijacking of the glucocorticoid receptor (GR) to substitute for the AR^[129]^. It is noteworthy that both the GR and AR bind to the same hormone response elements in the promoter of regulated genes^[130]^.

## Conclusion

It is apparent that intracrine androgen biosynthesis is an important component of resistance to ADT and ARSI inhibitors, and is clinically actionable. Knowledge of the nsSNPs that alter the activity or stability of the enzymes involved can determine who will benefit and who will not benefit from ARSI inhibitors. The enzyme that appears to be most important in mediating drug resistance is AKR1C3, and this is endorsed by the large number of small-molecule inhibitors that have been developed for this target both by academic laboratories and industry. Studies have moved beyond target validation and time will tell whether some of these compounds can transit preclinical development and make their way into well designed clinical trials for ARSI drug resistance. It is also apparent that AKR1C3 inhibitors do not necessarily have to be used as a monotherapy, but may improve progression-free survival in combination with other ARSIs.

## Declarations

### Copyright

© The Author(s) 2020.
